# Supplementary material for: Electrophysiological and Transcriptomic Features Reveal a Circular Taxonomy of Cortical Neurons
Source: Front Hum Neurosci. 2021 Jul 26;15:684950. doi: 10.3389/fnhum.2021.684950 (PMC8350032; doi:10.3389/fnhum.2021.684950)
Supplement: Supplementary file 1 [file Data_Sheet_1.pdf]

Electrophysiological and Transcriptomic Features Reveal a Circular Taxonomy of Cortical Neurons - *Supplementary Material*

The Supplementary Material is divided into four sections. The first section describes briefly the Machine Learning supervised methods considered in the paper, whereas the second section provides boxplots of the FMM model parameters by Cre line. The third section contains details about the different principal component analysis conducted to obtain the proposed circular taxonomy. The final section shows graphically the agreement between the proposed taxonomy and the results in (Tasic et al., 2016).

1 MACHINE LEARNING SUPERVISED METHODS

Short descriptions of the methods are given below, based on (Hastie et al., 2009) and (Izenman, 2008).

- **Linear discriminant analysis (LDA)**: supervised method which seeks the linear combination of features that maximizes the between-class variance relative to the within-class variance. It assumes that the probability functions conditioned to the class are distributed as multivariate normals with homoscedasticity. This simple and interpretable method performs well if the classes can be linearly separated. However, it may underfit the data, leading to high bias models that fail to capture patterns.
- **Random forest (RF)**: supervised method in which various decision trees, induced with random subsets of the input features, are aggregated under bagging. Decision trees discriminate observations of different classes by splitting the data into subsets based on the features values with criteria like the Gini impurity. In RF, the final class prediction is decided by majority voting of the underlying trees. It has become a widely used method due to its good results in many fields without requiring deep knowledge of the methodology by the user. RF provides directly feature importance measurements and while, it normally achieves a good balance between bias and variance, it occasionally overfits the data.
- **Gradient boosting decision trees (GBDT)**: supervised method in which 'weak' decision trees are induced sequentially to eventually lead to a final prediction based on a weighted majority vote. Classifiers are considered 'weak' when they make decisions slightly better than a random choice, meaning that their bias is high. Should the user be capable of a fine hyperparameter tuning, the results achieved in many complex problems are excellent. However, the data is overfitted sometimes.
- **Support vector machines (SVM)**: supervised method in which the maximal separating hyperplane between the classes is constructed in the input feature space. The procedure becomes more flexible by means of the 'kernel trick', which enlarges the features space to find nonlinear boundaries between classes. In particular, the SVM implementation used searched for polynomial boundaries. SVM are considered black box methods as their predictions cannot be easily reasoned. Regularization is often used as these models can overfit the data, resulting in models with high variance.
- **Model averaged neural network (AvNNet)**: supervised method in which multiple multi-layer perceptrons with different random weight initializations are trained to discriminate the classes in the data. Multi-layer perceptrons are neural networks in which data is fed forward through multiple hidden layers to extract useful and discriminating features. The relative lack of knowledge about the inherently extracted features as well as the reasoning behind their predictions make neural networks the most notorious black box method. Nevertheless, the accuracy of their predictions is often unrivaled. Data is frequently overfitted by these models, making essential regularization techniques such as weight decay.

2 BOXPLOTS OF THE FMM PARAMETERS BY CRE LINE

In this section, Figures S1-S5 depict boxplots of the main FMM parameters by Cre line. In these figures, the parameters corresponding to the representative neurons of the Cre line have been highlighted as stars.

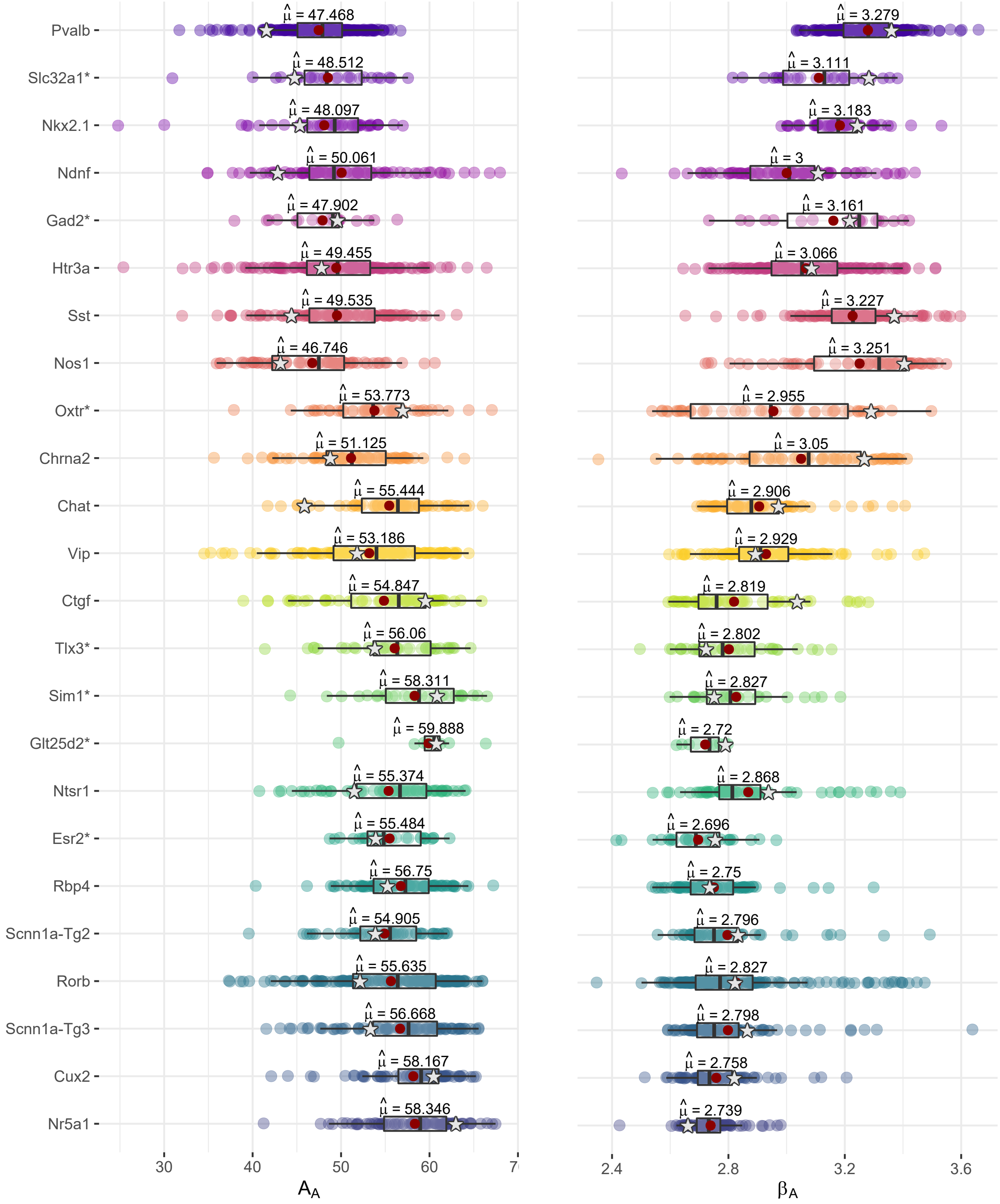

Figure S1. Distribution of the A_A and β_A parameters by Cre line. Parameters of representative neurons used throughout the manuscript are highlighted as stars.

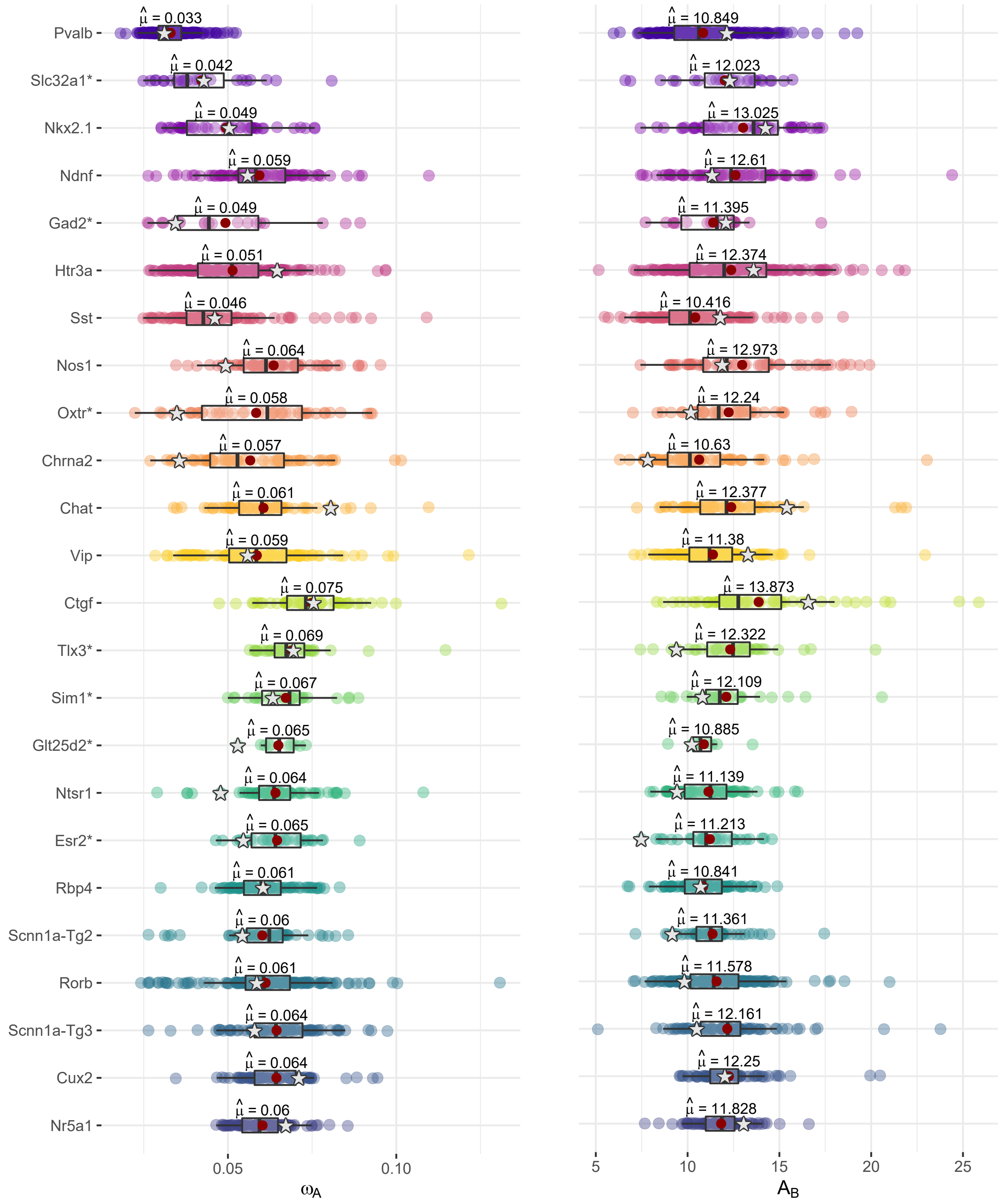

Figure S2. Distribution of the ω_A and A_B parameters by Cre line. Parameters of representative neurons used throughout the manuscript are highlighted as stars.

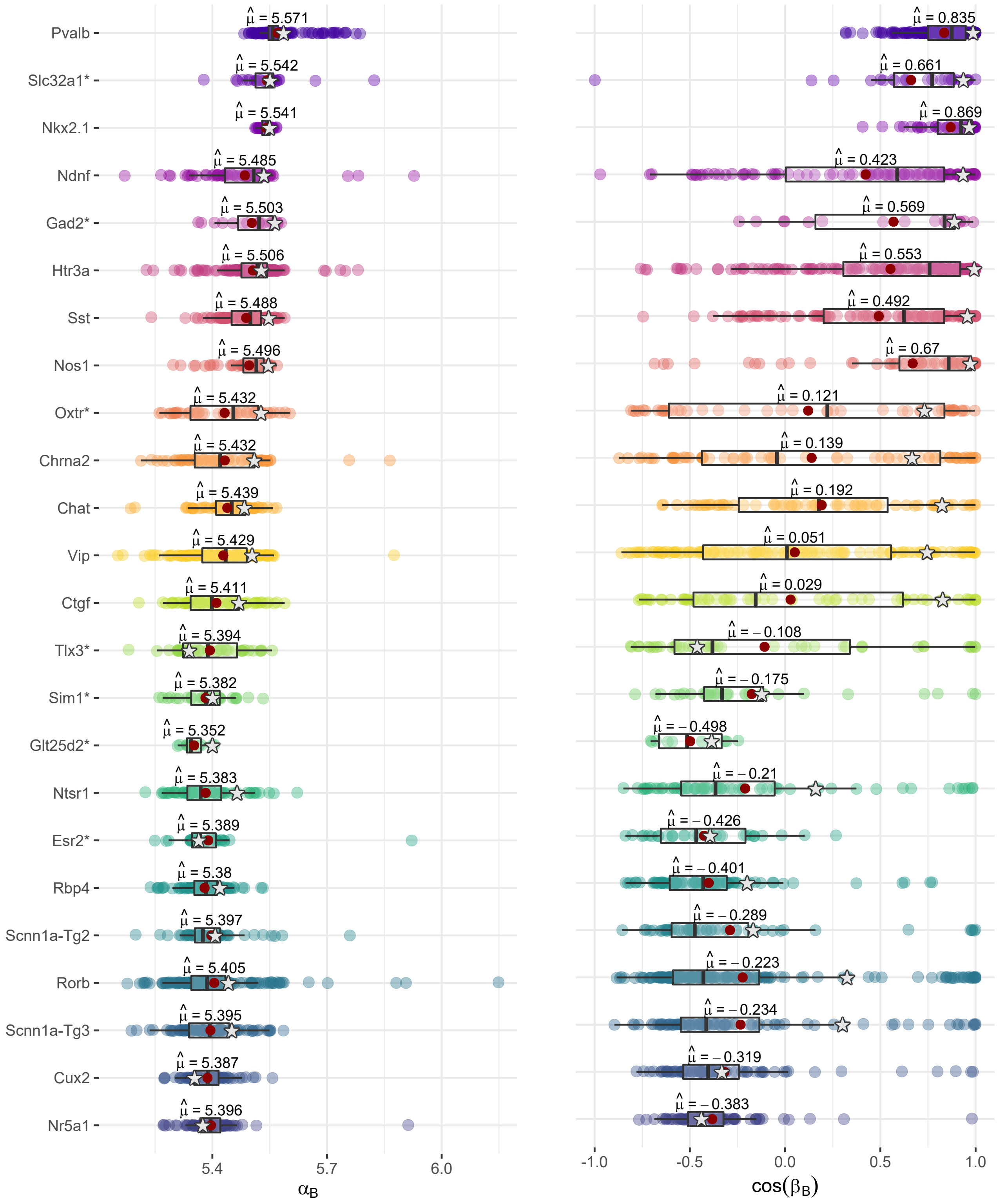

Figure S3. Distribution of the α_B and $\cos(\beta_B)$ parameters by Cre line. Parameters of representative neurons used throughout the manuscript are highlighted as stars.

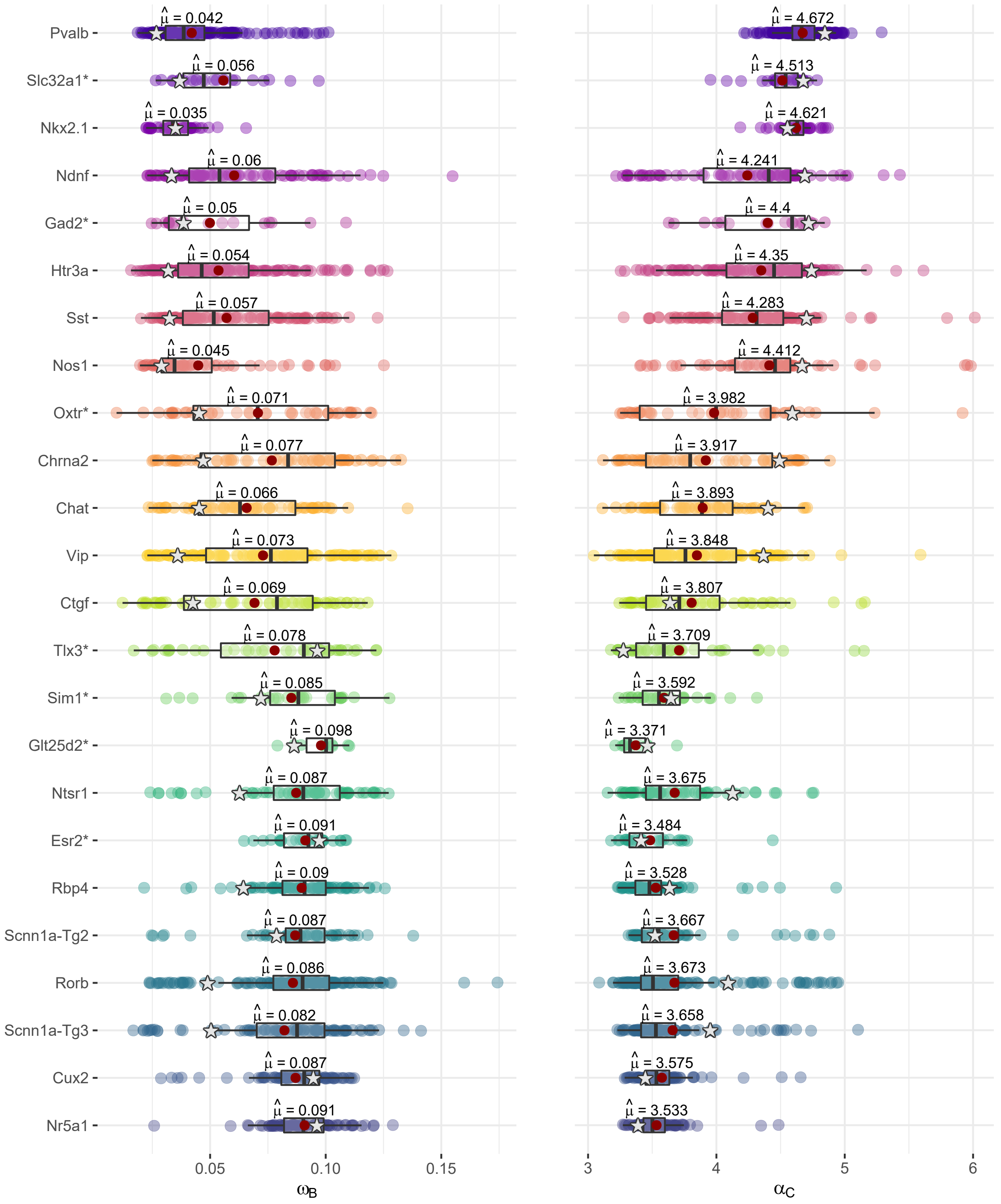

Figure S4. Distribution of the ω_B and α_C parameters by Cre line. Parameters of representative neurons used throughout the manuscript are highlighted as stars.

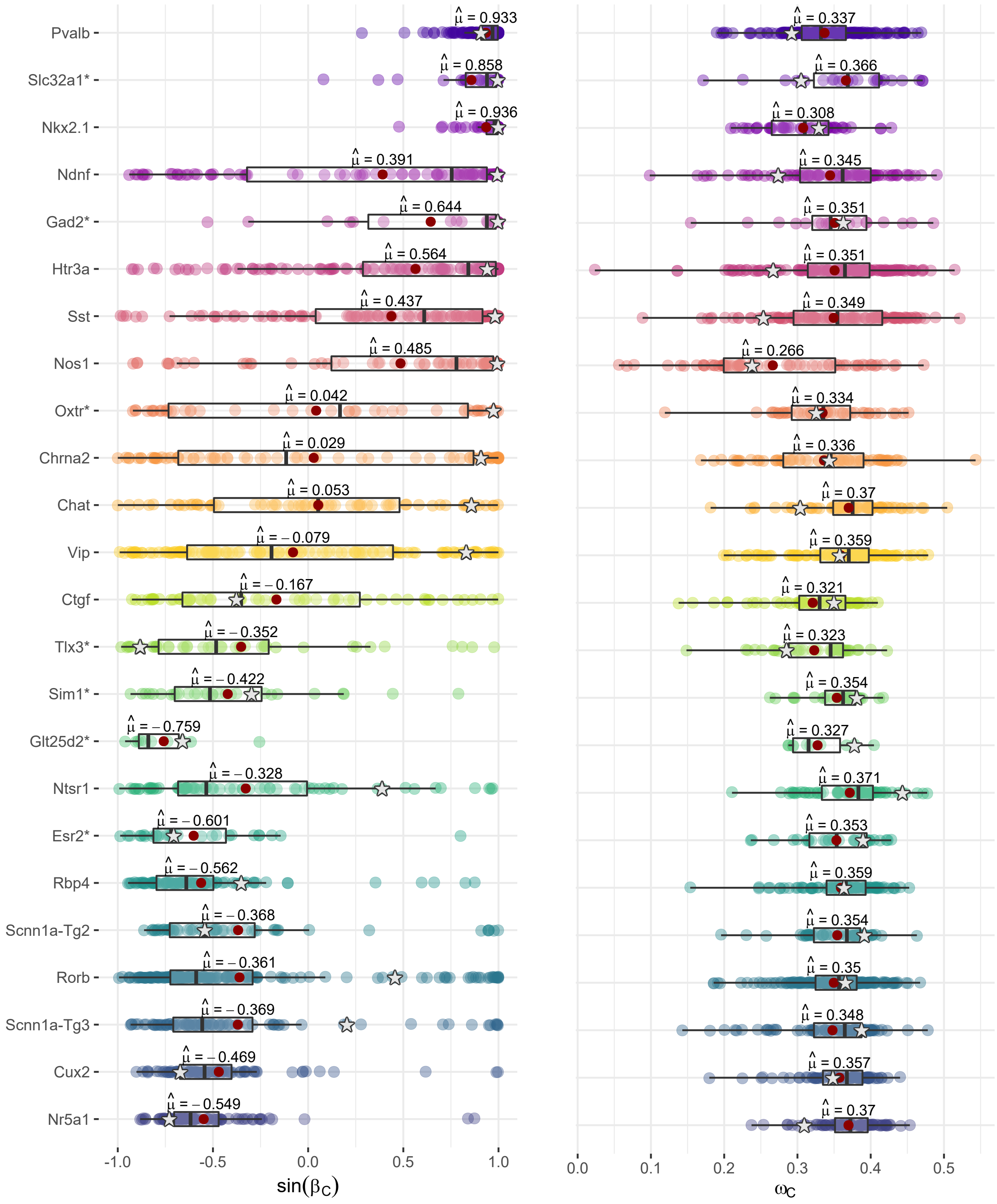

Figure S5. Distribution of the $\sin(\beta_C)$ and ω_C parameters by Cre line. Parameters of representative neurons used throughout the manuscript are highlighted as stars.

3 PRINCIPAL COMPONENT ANALYSIS (PCA)

This section illustrates in Figures S6-S10 different aspects of the three principal component analyses conducted in order to obtain the proposed circular taxonomy.

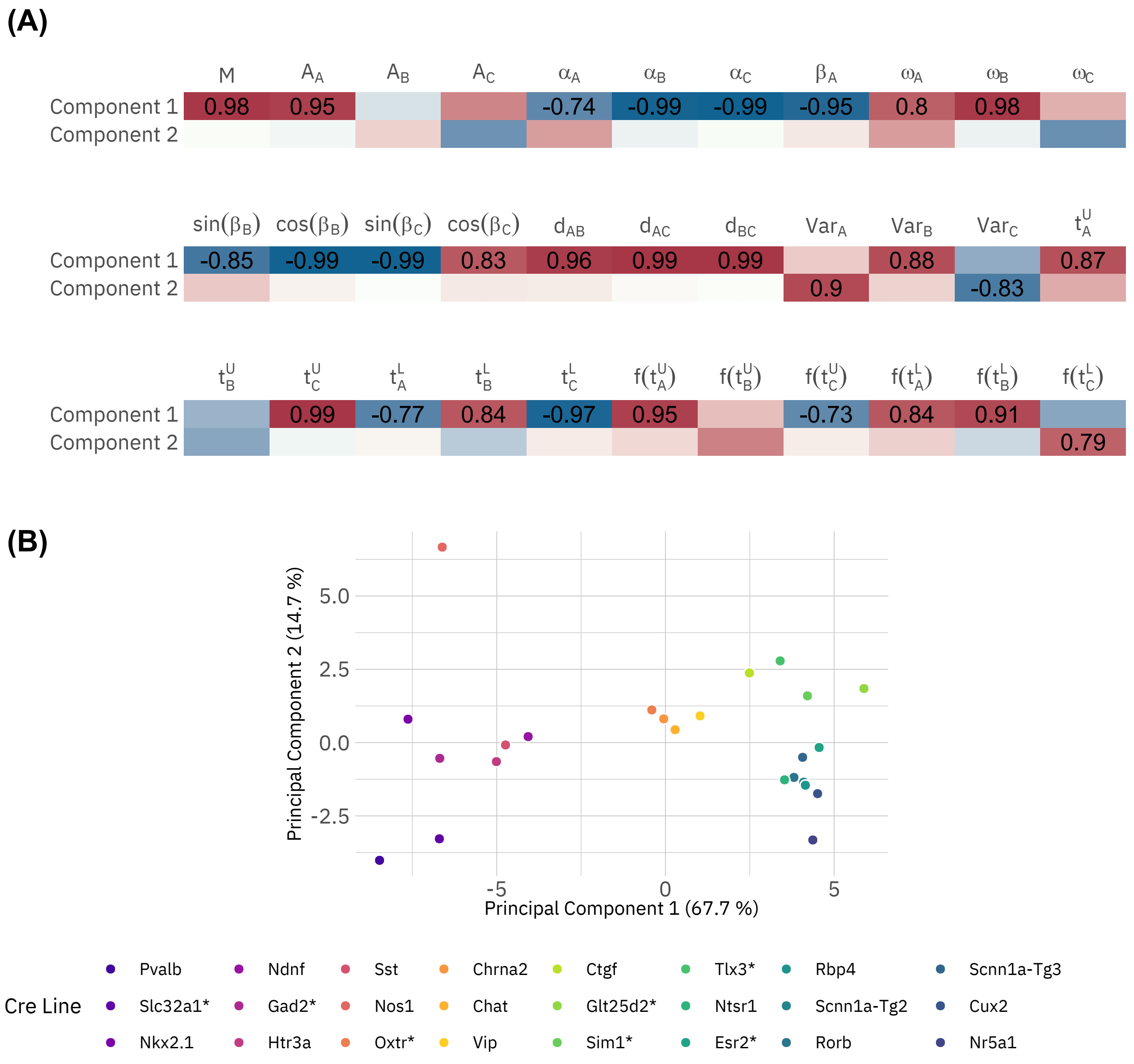

Figure S6. (A) Electrophysiological features correlation with extracted principal components. (B) Scores of the Cre lines with the electrophysiological features PCA.

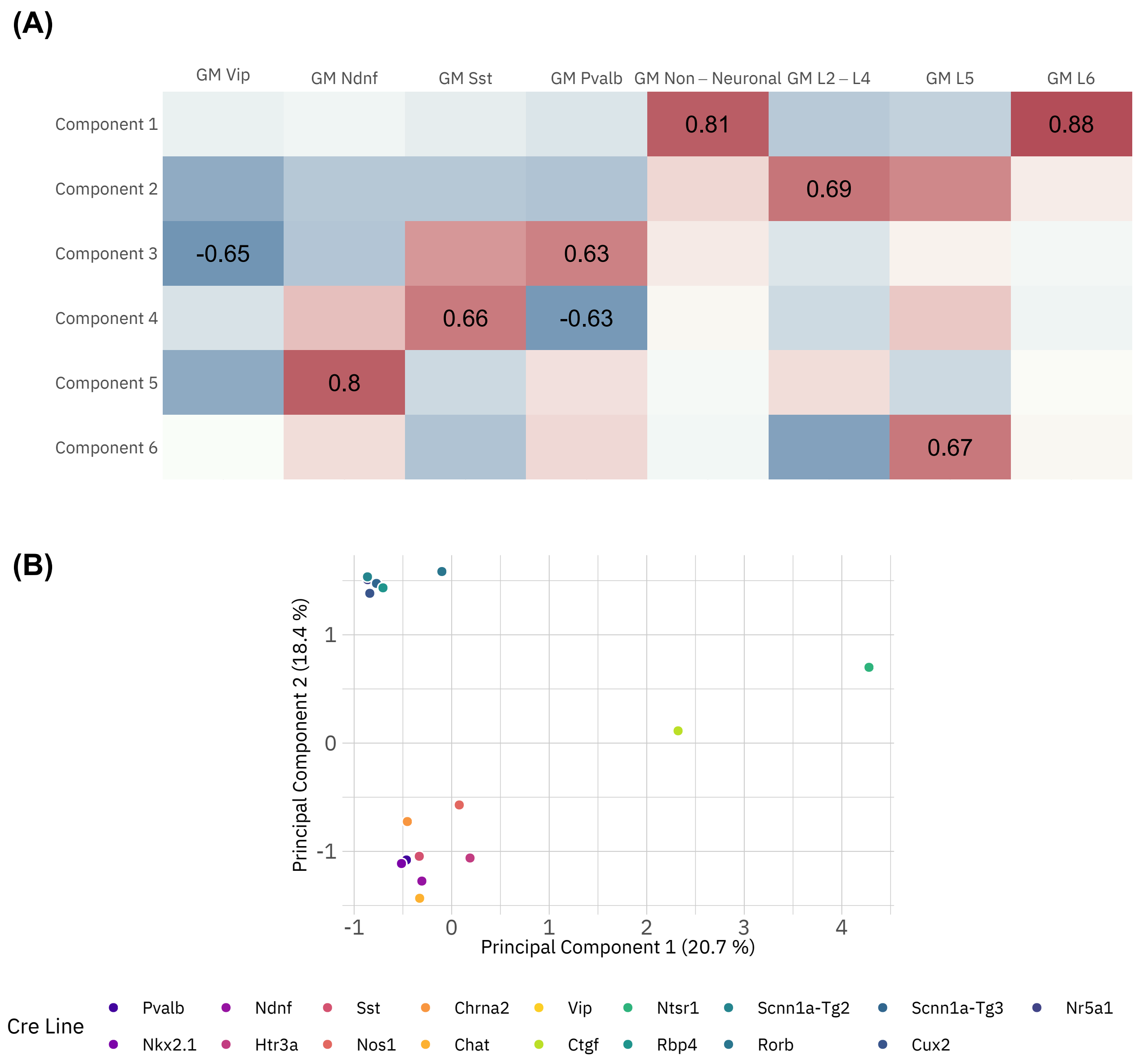

Figure S7. (A) Transcriptomic features correlation with extracted principal components. (B) Scores of the Cre lines with the transcriptomic features PCA.

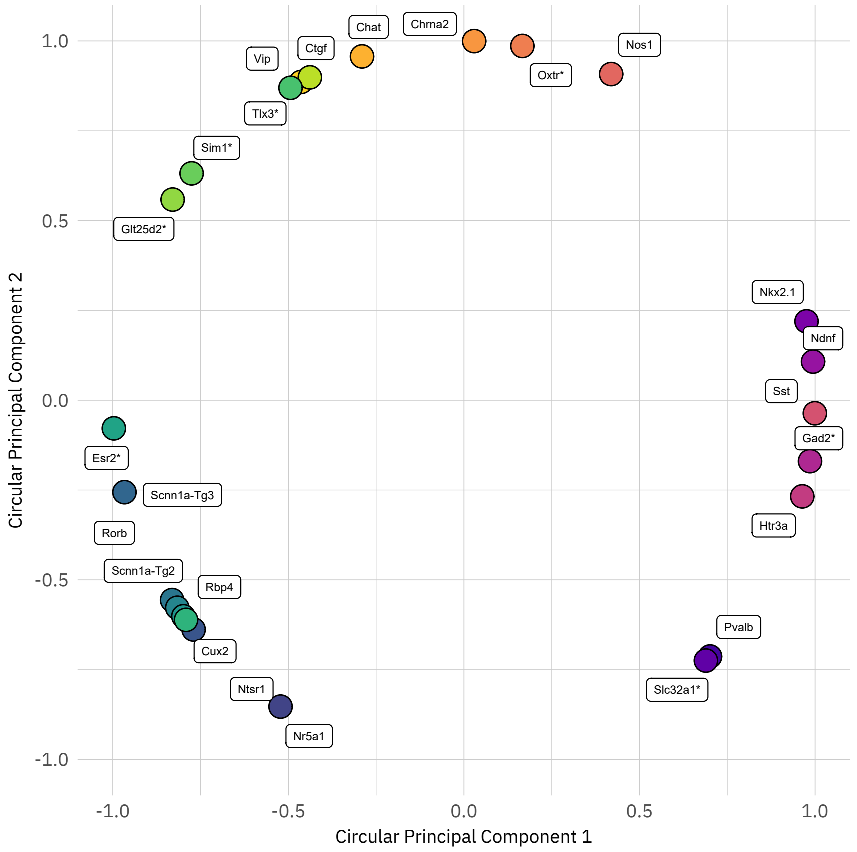

Figure S8. CPCA of the electrophysiological features.

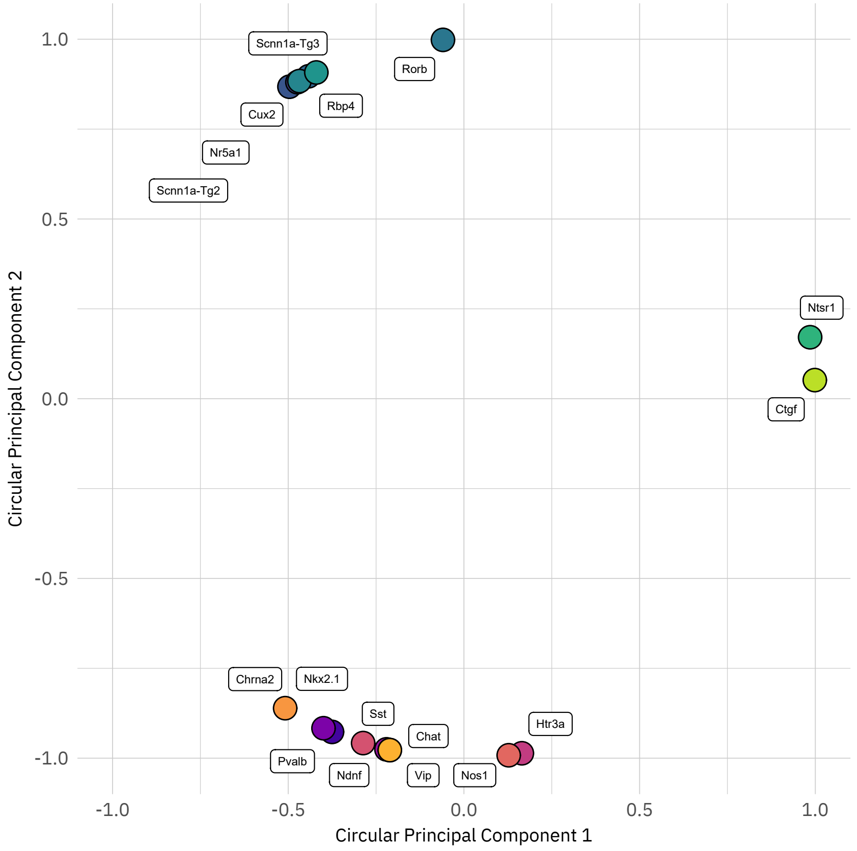

Figure S9. CPCA of the transcriptomic features.

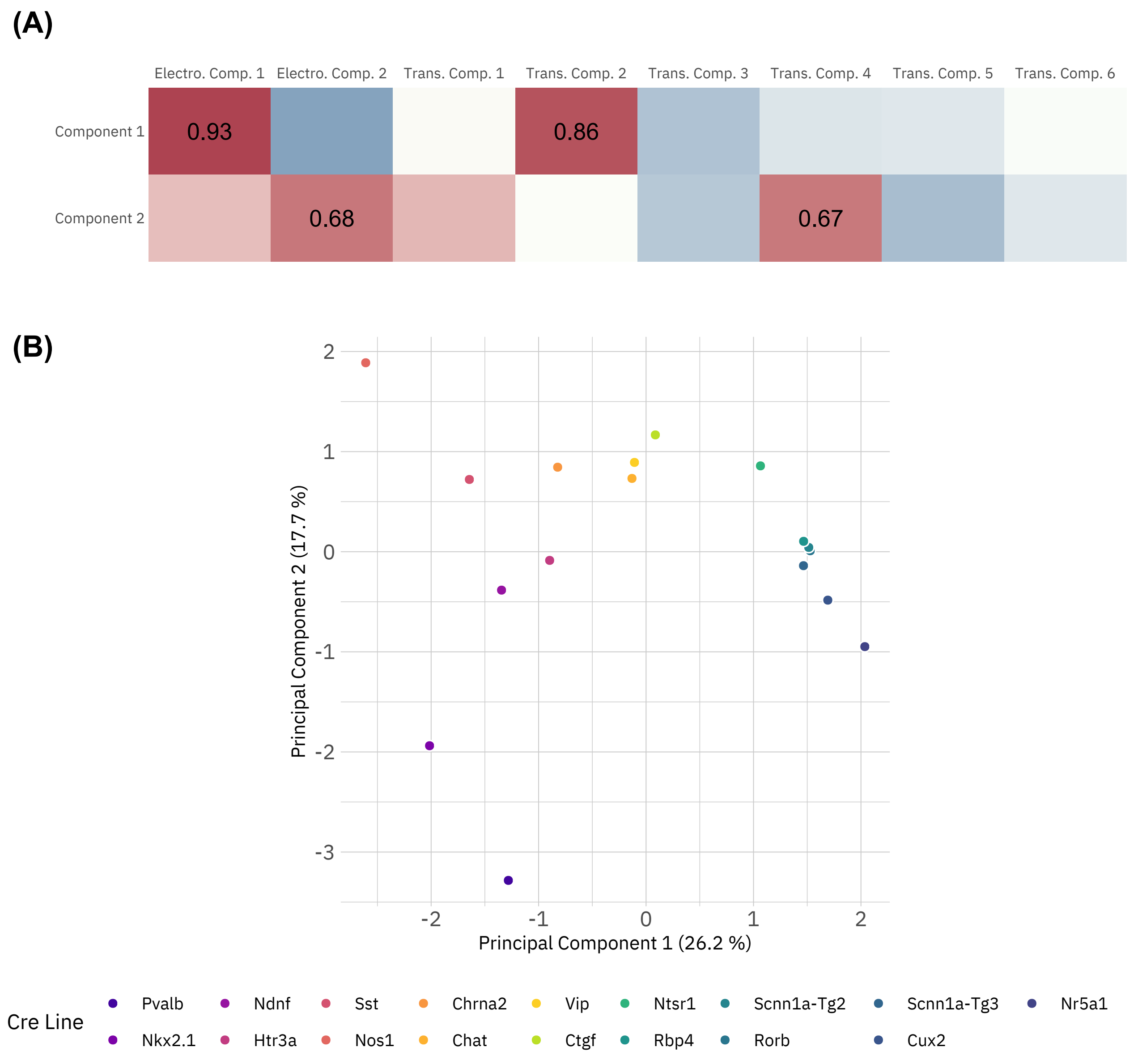

Figure S10. (A) Extracted electrophysiological and transcriptomic components correlation with extracted ensemble principal components. (B) Scores of the Cre lines with the ensemble PCA.

4 TRANSCRIPTOMIC CELL-TYPE MATRIX

Figure S11(A) shows the number of core cells by genetic cluster and Cre line from (Tasic et al., 2016). This Figure's rearrangement according to the proposed circular taxonomy order is shown on Figure S11(B).

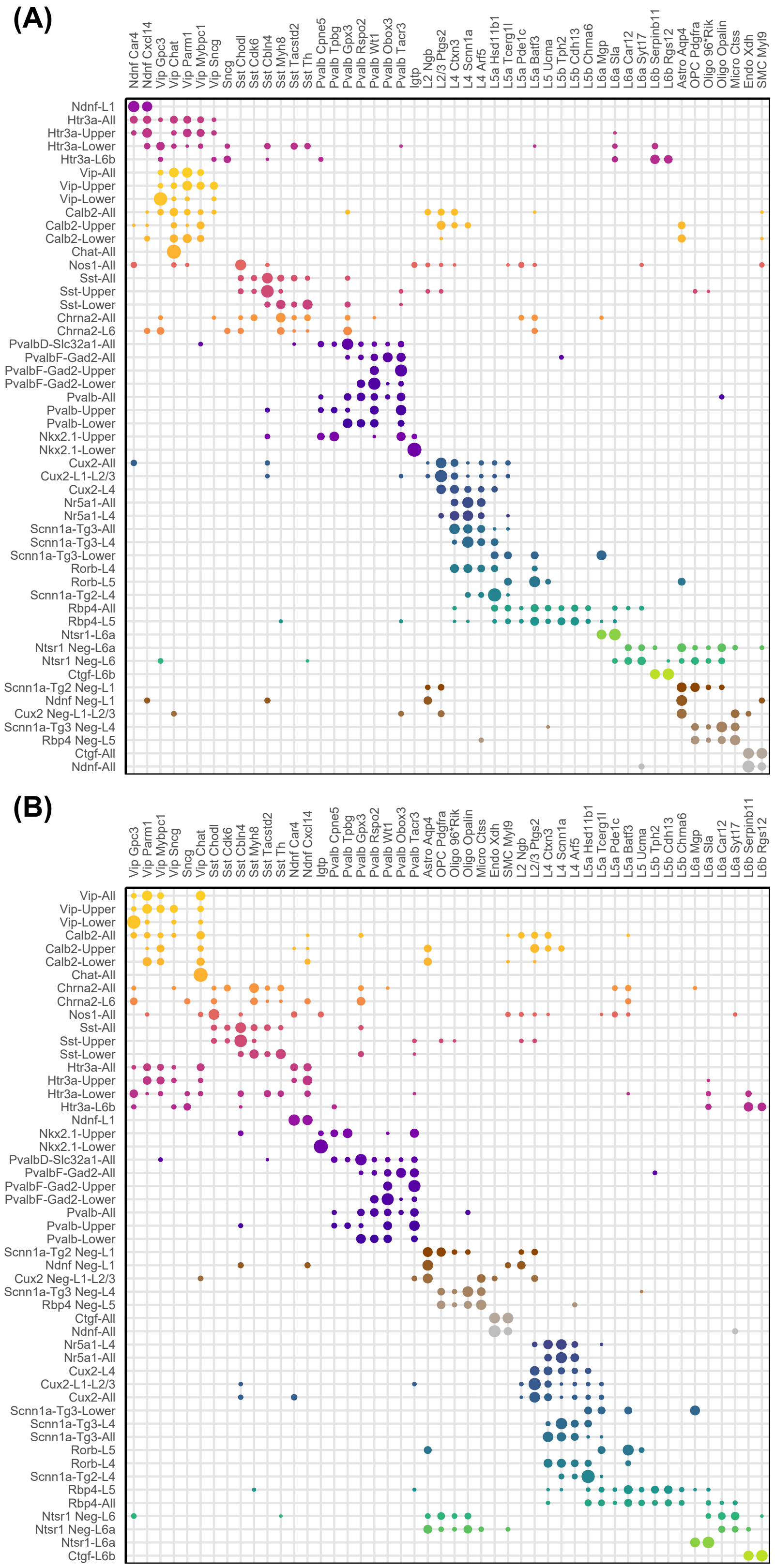

Figure S11. (A) Arrangement of the number of core cells by genetic cluster and Cre line according to (Tasic et al., 2016). (B) Rearrangement according to the proposed circular taxonomy.

REFERENCES

Hastie, T., Tibshirani, R., and Jerome, F. (2009). *The Elements of Statistical Learning: Data Mining, Inference, and Prediction* (Springer), second edn.

Izenman, A. J. (2008). *Modern Multivariate Statistical Techniques: Regression, Classification, and Manifold Learning* (Springer), first edn.

Tasic, B., Menon, V., Nguyen, T. N., Kim, S., Jarsky, T., Yao, Z., et al. (2016). Adult mouse cortical cell taxonomy revealed by single cell transcriptomics. *Nature Neuroscience* 19, 335–346
